# The RNA Degradation Pathway Regulates the Function of GAS5 a Non-Coding RNA in Mammalian Cells

**DOI:** 10.1371/journal.pone.0055684

**Published:** 2013-01-30

**Authors:** Hidenori Tani, Masaki Torimura, Nobuyoshi Akimitsu

**Affiliations:** 1 Research Institute for Environmental Management Technology, National Institute of Advanced Industrial Science and Technology (AIST), Tsukuba, Ibaraki, Japan; 2 Radioisotope Center, The University of Tokyo, Bunkyo, Tokyo, Japan; Keio University, Japan

## Abstract

Studies of various mRNAs have revealed that changes in the abundance of transcripts, through mRNA degradation, act as a critical step in the control of various biological pathways. Similarly, the regulation of non-coding RNA (ncRNA) levels is also considered to be important for their biological functions; however, far less is known about the mechanisms and biological importance of ncRNA turnover for the regulation of ncRNA functions. The growth arrest-specific 5 (GAS5) ncRNA accumulates during growth arrest induced by serum starvation and its transcript is degraded by the well characterized nonsense-mediated RNA decay (NMD) pathway. Historically, NMD was discovered as a RNA quality control system to eliminate aberrant transcripts; however, accumulating evidence shows that NMD also regulates the abundance of physiological transcripts. Interestingly, the GAS5 transcript has the ability to bind the glucocorticoid receptor (GR), resulting in the inhibition of its ligand-dependent association with DNA. The GR binds the promoters of various glucocorticoid-responsive genes, including apoptosis-related genes. In this study, we examined whether the RNA degradation pathway can regulate this function of GAS5. We measured the steady-state abundance and the decay rate of GAS5 in UPF1-depleted human cells using the 5′-bromo-uridine immunoprecipitation chase (BRIC) method, an inhibitor-free method for directly measuring RNA stability. We found that levels of the GAS5 transcript were elevated owing to prolonged decay rates in response to UPF1 depletion, and consequently the apoptosis-related genes, cIAP2 and SGK1, were down-regulated. In addition, serum starvation also increased the transcript levels of GAS5 because of prolonged decay rates, and conversely decreased levels of cIAP2 and SGK1 mRNA. Taken together, we found that the RNA degradation pathway can regulate the function of the GAS5 ncRNA in mammalian cells.

## Introduction

The regulation of mRNA degradation and stability in the cytoplasm has been well characterized. This regulation is a critical step in changing the abundance of certain transcripts controlling various biological pathways [1]–[3]. It has been estimated that 5%–10% of human genes are controlled at the level of RNA stability [4]. Indeed, there are different ways that mRNA stability is regulated. Each individual mRNA has its intrinsic stability under a given condition [5]–[7], and the stability of an individual mRNA may change in response to a variety of extracellular stimuli [8]–[10]. Similarly, the regulation of non-coding RNA (ncRNA) levels is considered important for their biological functions. ncRNAs can be roughly classified into two groups: small transcripts, such as microRNAs (miRNAs) [11], [12] and long transcripts, such as long intergenic non-coding RNAs (lincRNAs) [13], [14]. miRNAs are endogenous, small ncRNAs that play important regulatory roles in gene expression. Recent discoveries indicate that some miRNAs have a long half-life, but miRNA dynamics are important in specific cellular contexts, as well as the requirement of sequence elements for miRNA decay [15], [16]. In agreement with the varied life spans of mRNAs, long ncRNAs also have half-lives that vary. It was found that long ncRNAs with short half-lives included known regulatory ncRNAs, such as HOTAIR, CDKN2B-AS1/ANRIL, and NEAT1/MENε/β [7], [17]. Thus, it is expected there will be regulatory mechanisms for RNA degradation of long ncRNAs, as is the case for mRNA. However, little is known about the regulation of long ncRNAs.

Growth arrest-specific 5 (GAS5) is a long (∼650 bases in humans) ncRNA that was originally isolated from a screen for potential tumor suppressor genes expressed at high levels during growth arrest [18]. The human GAS5 gene is a multiple small nucleolar RNA (snoRNA) host gene that encodes 10 box C/D snoRNAs within 11 introns, and has been classified as a member of the 5′-terminal oligopyrimidine tract (5′ TOP) gene family, characterized by an upstream oligopyrimidine tract sequence [19]. The GAS5 transcript is increased during growth arrest induced by either serum starvation or treatment with translation inhibitors [19]–[22]. GAS5 has little protein-coding potential but its RNA is spliced, polyadenylated, and associates with ribosomes [19]. The GAS5 transcript is ubiquitously expressed, but it is unstable in human and murine proliferating cells [7], [17], [23]. The instability of GAS5 transcripts in proliferating cells can be explained by the phenomenon of nonsense-mediated RNA decay (NMD), whereby ribosome-associated GAS5 transcripts become targeted for degradation because of their premature stop codons [19]. Historically, NMD was discovered as a RNA quality control system to eliminate aberrant transcripts, growing evidences have shown that NMD also regulates the abundance of physiological transcripts [24]–[26]. Elimination of UPF1, an essential NMD factor, remarkably stabilizes the GAS5 transcript [25], [27]. Moreover, the overexpression of GAS5 causes both an increase in apoptosis induced by UV or cisplatin and a reduction in the rate of progression through the cell cycle. Consistently, the downregulation of endogenous GAS5 inhibits apoptosis and maintains a more rapid cell cycle, indicating that GAS5 expression is both necessary and sufficient for normal growth arrest in human cells, and it suggests that GAS5 can act as a tumor suppressor [28], [29]. Recently, Kino et al. reported that GAS5 functions as a starvation- or growth arrest-linked riborepressor for the glucocorticoid receptor (GR) by binding to the DNA-binding domain of the GR, acting as a decoy glucocorticoid response element (GRE), thus competing with DNA GREs for binding to the GR [30]. This results in the inhibition of the ligand-dependent association of the GR with GREs of various glucocorticoid-responsive genes including apoptosis-related genes.

In this study, we examined whether the NMD RNA degradation pathway regulates the function of GAS5 as a model long ncRNA regulation. RNA decay has been assessed by blocking global transcription with traditional transcriptional inhibitors such as actinomycin D (ActD), 5,6-Dichloro-1-β-D-ribofuranosyl-benzimidazole (DRB), and α-amanitin (α-Am) [1], [31]. However, inhibitor-mediated global transcriptional arrest has a profound impact on cellular physiology, and affects RNA decay rates [7], [23], [32]. Thus, we used a novel inhibitor-free method of pulse-labeling nascent transcripts with 5′-bromouridine [7], [26], [33] or with 5′-ethynyluridine [33]–[36] to enable the measurement of RNA decay under non-disruptive conditions. We found that GAS5 levels were elevated owing to prolonged decay rates in response to UPF1 depletion, and that multiple apoptosis-related genes were downregulated. Similarly, serum starvation increased the expression level of GAS5 because of prolonged decay rates, and decreased the mRNA levels of apoptosis-related genes.

## Materials and Methods

### Cell culture

HEK293T and HEK293 cells were grown in Dulbecco's modified Eagle's medium (DMEM), supplemented with 10% fetal bovine serum (FBS) and antibiotics at 37°C in a humidified incubator with 5% CO_2_.

### siRNA treatments

The sequences of the siRNAs were: control siRNA (sense: 5′-GUACCUGACUAGUCGCAGAAG-3′, antisense: 5′-GUACCUGACUAGUCGCAGAAG-3′), UPF1 siRNA (sense: 5′-GAUGCAGUUCCGCUCCAUUdTdT-3′, antisense: 5′-AAUGGAGCGGAACUGCAUCdTdT-3′), UPF1 siRNA (2) (sense: 5′-AAUUUCUGUAACUUGUUUCCU-3′, antisense: 5′-GAAACAAGUUACAGAAAUUAC-3′), and GAS5 siRNA (sense: 5′-CUUGCCUGGACCAGCUUAAUU-3′, antisense: 5′-UUAAGCUGGUCCAGGCAAGUU-3′). These siRNAs were transfected into cells using Lipofectamine RNAiMAX (Invitrogen), according to the manufacturer's instructions. siRNA duplexes were used at a final concentration of 10 nM. Cells were harvested 48 h after transfection and total RNAs were isolated using RNAiso Plus (Takara), according to the manufacturer's instructions. RT-qPCR analysis was used to determine whether RNA interference achieved significant depletion of each target sequence.

### Over-expression of GAS5

A GAS5 (1−631) expression vector was constructed by subcloning the full-length GAS5 sequence lacking a poly A tail (based on the GAS5 sequence, NR_002578, in NCBI). The GAS5 cDNA was amplified from total RNA purified from HeLa cells, and then cloned into pcDNA3.1 (+) (Invitrogen). Additional information on vector construction can be provided upon request. The expression vector, at 1 µg/mL, was transfected into cells using Lipofectamine 2000 (Invitrogen), according to the manufacturer's instructions. Cells were harvested 48 h after transfection and total RNAs were isolated using RNAiso Plus (Takara), according to the manufacturer's instructions. RT-qPCR analysis was used to determine the levels of GAS5 over-expression.

### Reverse Transcription - quantitative real-time Polymerase Chain Reaction (RT-qPCR)

The isolated RNA was reverse transcribed into cDNA using the PrimeScript RT Master Mix (Perfect Real Time) (Takara). The cDNA was amplified using the following primer sets: GAPDH (forward: 5′-GCACCGTCAAGGCTGAGAAC-3′, reverse: 5′-TGGTGAAGACGCCAGTGGA-3′), ACTB (forward: 5′-CCAACCGCGAGAAGATGA-3′, reverse: 5′-CCAGAGGCGTACAGGGATAG-3′), GAS5 (forward: 5′-CTTGCCTGGACCAGCTTAAT-3′, reverse: 5′-CAAGCCGACTCTCCATACCT-3′), UPF1 (forward: 5′-AGATCACGGCACAGCAGAT-3′, reverse: 5′-TGGCAGAAGGGTTTTCCTT-3′), MALAT1 (forward: 5′-GCTGTGGAGTTCTTAAATATCAACC-3′, reverse: 5′-TTCTCAATCCTGAAATCCCCTA-3′), cIAP2 (forward: 5′-TCTAGTGTTCTAGTTAATCC-3′, reverse: 5′-ACCACTTGGCATGTTGAACC-3′), SGK1 (forward: 5′-CTATGCTGCTGAAATAGC-3′, reverse: 5′-GTCCGAAGTCAGTAAGG-3′), ADRP (forward: 5′-CTGTGGCCAGCACGATCAC-3′, reverse: 5′-CTCACGAGCTGCATCATC-3′), GADD45A (forward: 5′-TTTGCAATATGACTTTGGAGGA-3′, reverse: 5′-CATCCCCCACCTTATCCAT-3′), SMG1 (forward: 5′-TCGAAGTCAAGAACACGTTGA-3′, reverse: 5′-GGGTGATGCAAAACTCACTAAA-3′), EIF4A3 (forward: 5′-GGATGAAGCTGATGAAATGTTG-3′, reverse: 5′-TGGTCATCTCCAGAATCTCGT-3′), and CASC3 (forward: 5′-GGGGTTCCAGTTAATACAAGTTTC-3′, reverse: 5′-GCCAGCTGTATTTCTCTTCTGAG-3′). GAPDH and ACTB were used for normalization. SYBR Premix Ex Taq II (Perfect Real Time) (TaKaRa) or THUNDERBIRD SYBR qPCR mix (TOYOBO) was used according to the manufacturer's instructions. RT-qPCR analysis was performed using a Thermal Cycler Dice Real Time System (Takara) or MyiQ2 (BIO-RAD).

### BRIC

BRIC was performed as previously described [7], [26], [33]. In brief, cells were incubated at 37°C in the presence of 150 µM 5′-bromo-uridine (BrU) (Wako) for 24 h in a humidified incubator with 5% CO_2_. At indicated time points after replacing BrU-containing medium with BrU-free medium, cells were harvested. Total RNA was isolated using RNAiso Plus (Takara). Two micrograms of BrU-labeled total RNA were denatured by heating at 80°C for 1 min and then added to 2 µg of anti-BrdU mAb-conjugated beads (clone 2B1, MBL). The mixture was incubated at room temperature for 1 h with rotation. Beads were washed three times with 0.1% BSA in PBS. ISOGEN LS (Nippon Gene) was added, followed by RNA isolation in accordance with the manufacturer's instructions. The isolated RNA was used for RT-qPCR.

### 5-Ethnyluridine (EU) pulse-labeling

Analysis of RNA half-lives was performed by EU pulse-labeling of RNA using the Click-iT Nascent RNA Capture Kit (Invitrogen) [33]–[36]. After serum withdrawal for 24 h, 200 µM of EU was added to medium and cells incubated for an additional 24 h. At indicated time points after replacing EU-containing medium with EU-free medium, cells were harvested. Total RNA was isolated using RNAiso Plus (Takara). Then, EU-labeled RNAs were biotinylated and captured using the Click-iT Nascent RNA Capture Kit (Invitrogen), according to the manufacturer's instructions. Isolated RNA s were used for RT-qPCR.

## Results

### Half-life of GAS5 RNA

**Figure 1:**
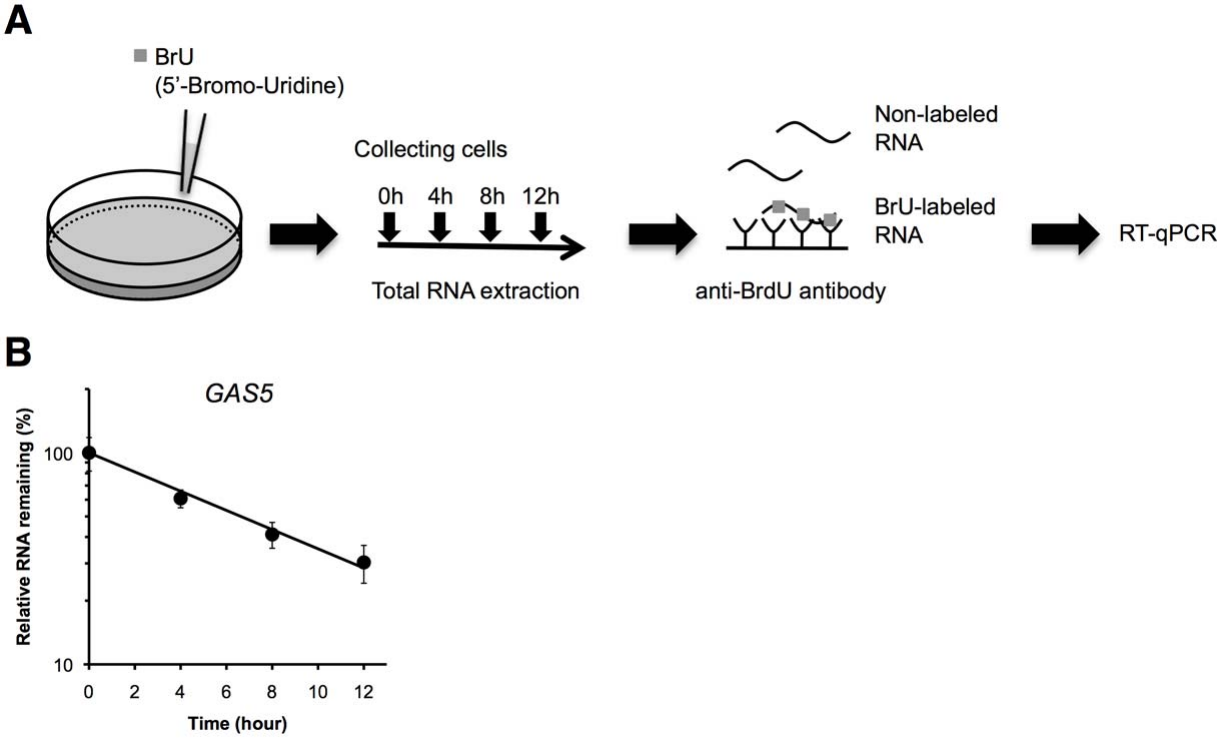
Decay rate of GAS5. (A) Schematic representation of 5′-Bromo-uridine immunoprecipitation chase (BRIC). BrU is incorporated into newly transcribed RNA in place of uridine. Cells are collected and total RNA extracted at multiple time points after BrU washout. BrU-labeled RNAs are immunopurified using anti-BrdU beads. Non-labeled RNAs are discarded. Then, BrU-labeled RNAs are quantified by RT-qPCR. (B) The decay rate of GAS5 as determined by BRIC in HEK293T cells. Relative quantitative values at time 0 h were set to 100%. Values represent mean ± errors obtained from two independent experiments.

We first determined the decay rate of GAS5 in HEK293T cells using the BRIC system [7], [26], [33]. In the BRIC system (Figure 1A), BrU-labeled RNAs were immunopurified from total RNA isolated from HEK293T cells at four time points after BrU pulse-labeling. Using this method we found the half-life of GAS5 in HEK293T cells to be 6.6 h (Figure 1B). A previous study using the BRIC system reported that the half-life of GAS5 in HeLa cells was 2.6 h [7]. The half-life of GAS5 in HEK293T cells is relatively long compared with that in HeLa cells.

### GAS5 transcripts are degraded by the NMD pathway

**Figure 2:**
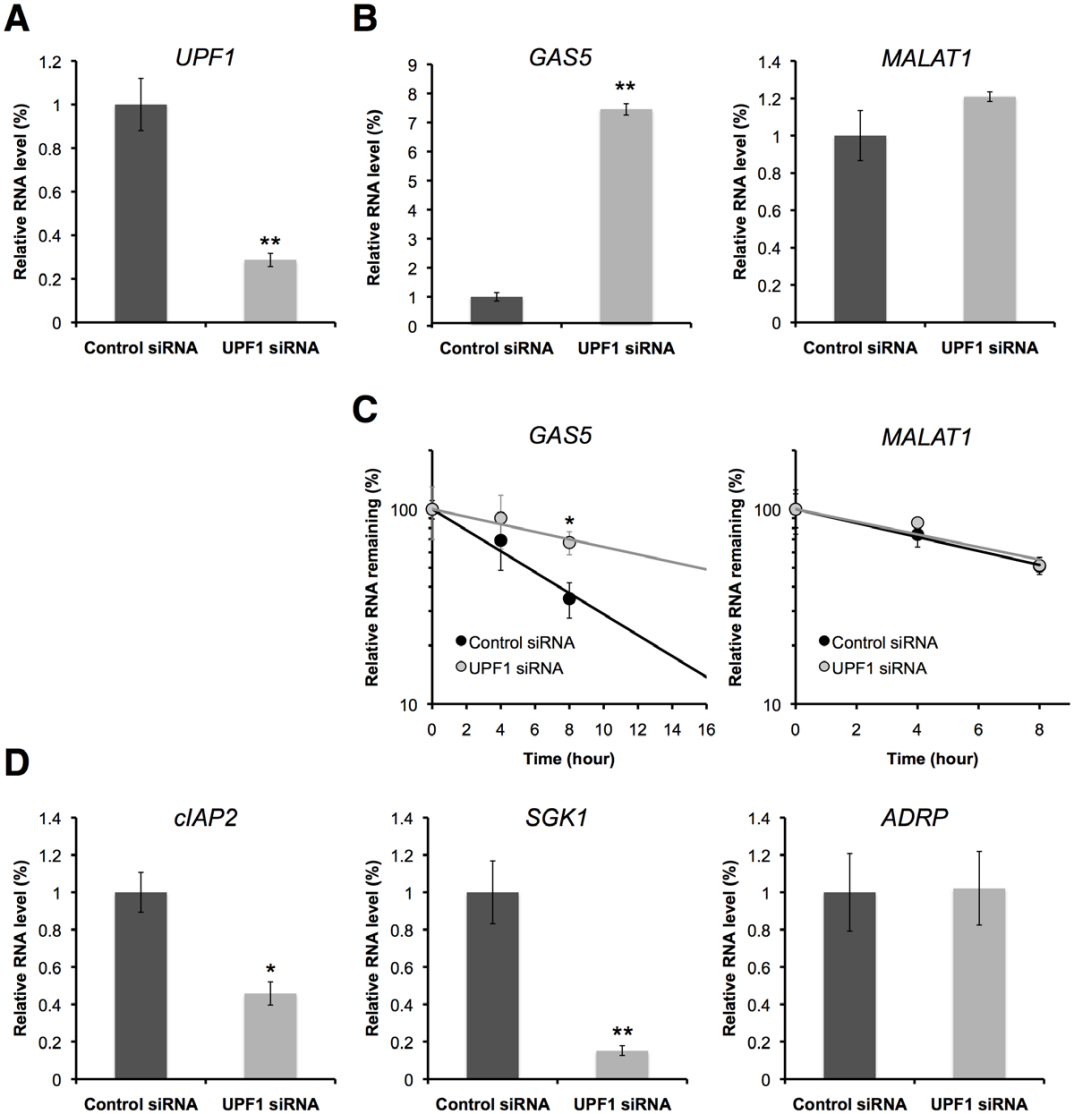
Knockdown of UPF1 increases the expression level and prolongs the decay rate of GAS5, and decreases the expression of glucocorticoid-responsive genes. HEK293T cells were treated with a control siRNA or with a siRNA targeting UPF1. (A) The expression levels of UPF1 in control cells (black bar) and in UPF1-depleted cells (grey bar) were determined by RT-qPCR. (B) The expression levels of GAS5 and MALAT1 in control cells (black bar) and in UPF1-depleted cells (grey bar) were determined by RT-qPCR. (C) The decay rates of GAS5 and MALAT1 were determined by BRIC in control cells (solid circle and black bar) and in UPF1-depleted cells (open circle and grey bar). Relative quantitative values at 0 h were set to 100%. (D) The expression levels of glucocorticoid-responsive genes, cIAP2 and SGK1, and the PPARδ-responsive gene, ADRP, in control cells (black bar) and in UPF1-depleted cells (grey bar) were determined by RT-qPCR. (A–D) The levels of GAPDH were used for normalization. Values represent mean±SD obtained from three independent experiments (*P<0.05, **P<0.01, Student's t test).

To examine whether GAS5 transcripts are targeted for NMD, we determined the steady-state abundance and the decay rate of GAS5 with an abrogated NMD pathway by UPF1 depletion. Depletion of UPF1 by RNA interference (RNAi), using two different siRNAs to rule out off-target effects, reduced UPF1 mRNA levels in HEK293T cells to ∼28.6% of the control level (Figure 2A, S1). Knockdown of UPF1 increased the steady-state level of GAS5 by about ∼7.5 fold (Figure 2B, S1). This result is consistent with previous reports [25], [27]. Moreover, its half-life (*t*_1/2_) was prolonged from 5.6 h to 15.4 h in UPF-depleted cells (Figure 2C). The half-life of GAS5 in the control siRNA-treated cells (*t*_1/2_ = 5.6 h in Figure 2C) differed from that in normal cells (*t*_1/2_ = 6.6 h in Figure 1B). This difference is probably due to differences in cell culture conditions, e.g. addition of siRNAs and transfection reagents to siRNA-treated cells but not to normal cells. In contrast, knockdown of UPF1 did not affect the steady-state level or decay rate of MALAT1 [37]–[41], another nuclear long ncRNA (Figure 2B, 2C, S1). Moreover, the abundance of NMD-sensitive transcripts, GADD45A and SMG1, were significantly upregulated in response to UPF1 depletion, while those of non-NMD-sensitive transcripts, EIF4A3 and CASC3, were unchanged (Figure S1) [26]. These results validate UPF1 depletion for effective inhibition of the NMD pathway. Thus, we concluded that GAS5 is specifically degraded by the NMD pathway, and that NMD can regulate the abundance of a GAS5.

### The abundance of GAS5 modulates the transcriptional activity of apoptosis-related genes

**Figure 3:**
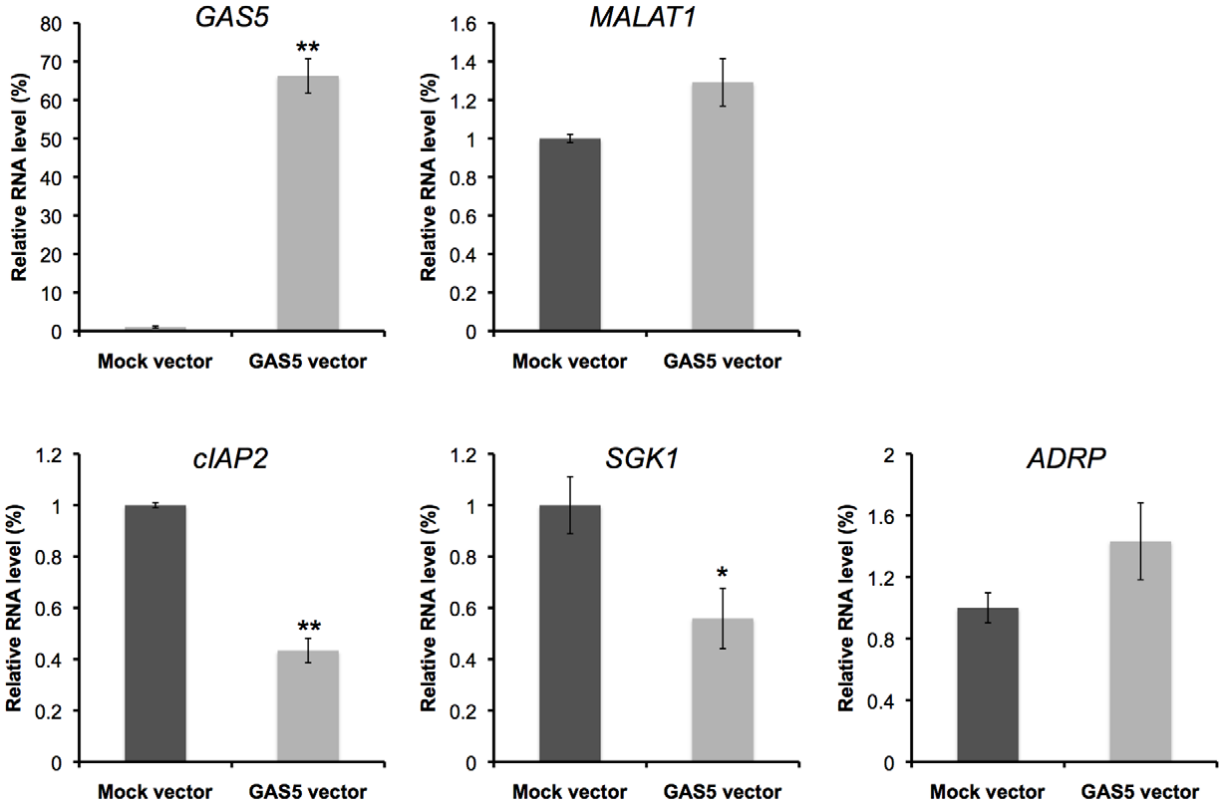
Over-expression of GAS5 decreased the expression levels of glucocorticoid-responsive genes. HEK293 cells were treated with plasmid vectors as indicated. The expression levels of the indicated RNAs in control cells with mock vector (black bar) and in cells over-expressing GAS5 (grey bar) were determined by RT-qPCR. The levels of GAPDH and ACTB were used for normalization. Values represent mean±SD obtained from three independent experiments (*P<0.05, **P<0.01, Student's t test).

GAS5 modulates the transcriptional activity of the glucocorticoid receptor and acts as a sensitizer of apoptosis by suppressing glucocorticoid-mediated induction of several responsive genes, including the cellular inhibitor of apoptosis 2 (cIAP2) and serum- and glucocorticoid-regulated kinase 1 (SGK1) [30]. The cIAP2 and the SGK1 are apoptosis inhibitors [42], [43]. The cIAP2 protein was found to bind caspases 3, 7, and 9 and inhibit their activities, thereby suppressing apoptosis triggered by treatment activation of the Fas receptor or the tumor necrosis factor–α (TNF–α) [42]. The cIAP2 gene promoter has imperfect tandem GREs ∼500 bp upstream of its transcriptional initiation site and GR activates its transcription by binding to these GREs [44], [45]. SGK1 also contains functional GREs in its promoter region [46]. To examine whether the mRNA levels of cIAP2 and SGK1 are decreased by the accumulation of GAS5, their mRNA levels were quantified in the cells where GAS5 was stabilized by the depletion of UPF1. GAS5 levels increased up to 7.5 fold in UPF1 knocked-down cells (Figure 2B, S1) and expectedly, the mRNA levels of cIAP2 and SGK1 were significantly decreased in the UPF1 knocked-down cells (Figure 2D, S1). In contrast, the mRNA level of the peroxisome proliferator–activated receptor δ (PPARδ)-responsive gene, adipose differentiation-related protein (ADRP) [47], whose transcription is not regulated by GAS5, was not significantly changed (Figure 2D, S1). We have also examined whether over-expression of GAS5 produces opposite effects to those of UPF1 knockdown. Consistent with the expectation from the knockdown experiments, GAS5 over-expression specifically down-regulated the expression levels of cIAP2 and SGK1 (Figure 3). These results suggest that the NMD pathway can regulate the levels of GAS5, resulting in a change in the transcriptional activity of apoptosis-related genes.

### Serum starvation increases the expression level of GAS5 and decreases the GR-responsive genes

**Figure 4:**
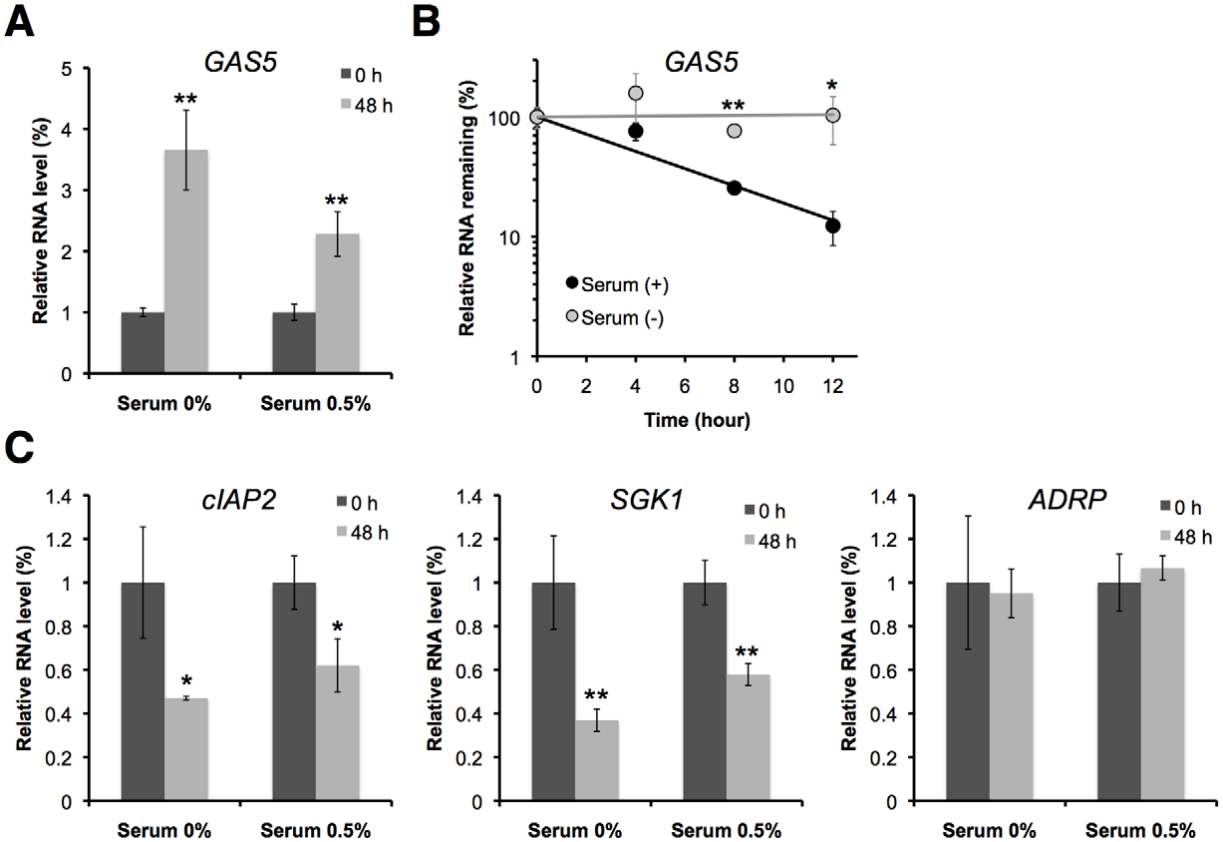
Serum starvation increases the expression level and prolongs the decay rate of GAS5, and decreases the expression of glucocorticoid-responsive genes. HEK293 cells were cultured with 0, 0.5, and 10% serum as indicated. (A) The relative expression levels of GAS5 in cells with 0 and 0.5% serum relative to control cells (cultured with 10% serum) were determined by RT-qPCR. (B) The decay rates of GAS5 were determined using the Click-iT Nascent RNA Capture Kit (Invitrogen) in control cells (solid circle and black bar) and in serum-starved cells (open circle and grey bar). Relative quantitative values at 0 h were set to 100%. (C) The relative expression levels of glucocorticoid-responsive genes, cIAP2 and SGK1, and the PPARδ-responsive gene, ADRP, in cells with 0 and 0.5% serum relative to control cells (cultured with 10% serum) were determined by RT-qPCR. (A–C) The levels of GAPDH and ACTB were used for normalization. Values represent mean±SD obtained from two (in B) or three (in A and C) independent experiments (*P<0.05, **P<0.01, Student's t test).

**Figure 5:**
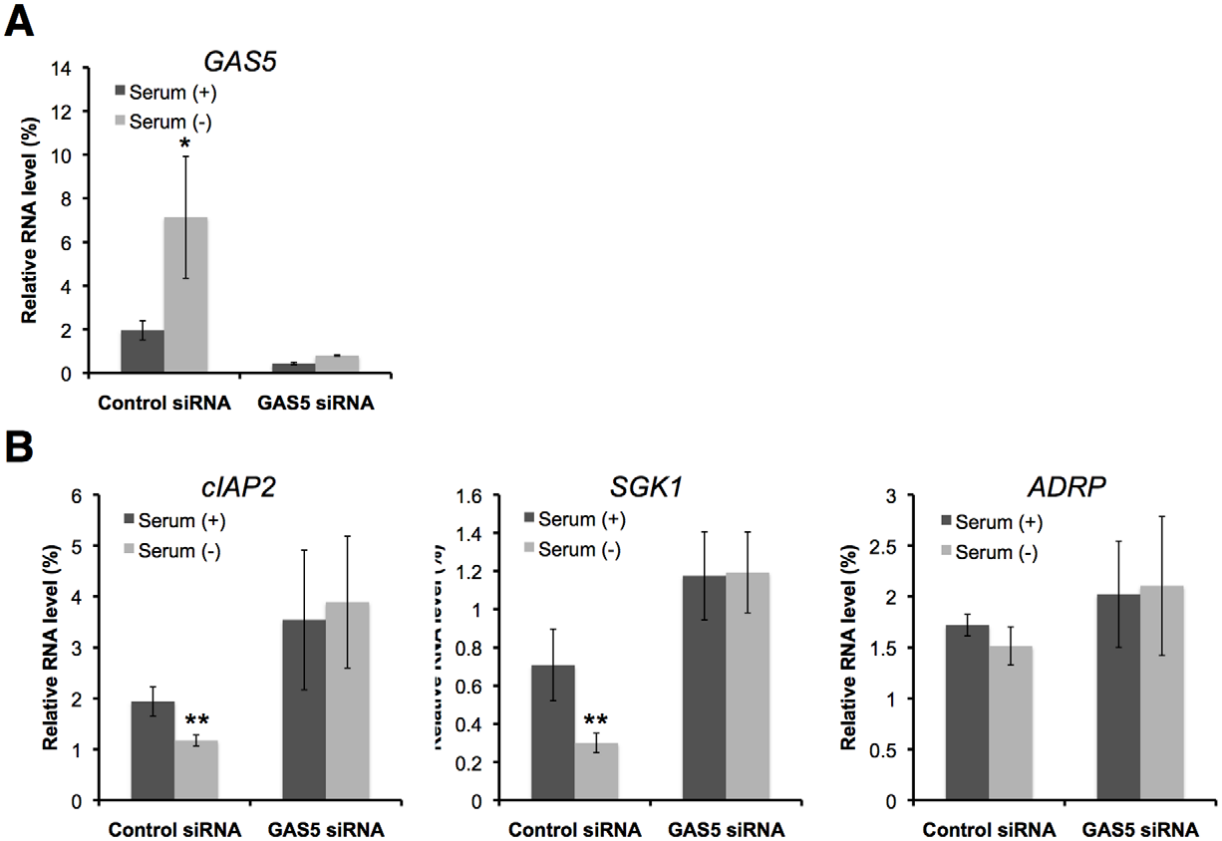
Knockdown of GAS5 suppresses the effects of serum withdrawal on the expression of glucocorticoid-responsive genes. HEK293 cells were treated with a control siRNA or with a siRNA targeting GAS5, and cultured either with or without serum for 48 h. (A, B) The relative expression levels of the indicated RNAs in cells with serum (black bar) or without serum (grey bar) relative to control (cells cultured at 0 h) were determined by RT-qPCR. The levels of GAPDH and ACTB were used for normalization. Values represent mean±SD obtained from three independent experiments (*P<0.05, **P<0.01, Student's t test).

GAS5 is a 5′ TOP class gene [19]. Growth arrest by serum starvation is associated with attenuated translation of the 5′ TOP transcripts and inhibition of their degradation, resulting in accumulation of the GAS5 transcript [19]–[22]. To examine whether the mRNA levels of the GR-responsive genes, cIAP2 and SGK1, are decreased by the accumulation of GAS5 upon serum starvation, we cultured HEK293 cells in serum-free medium or in medium with a small amount of serum (0.5%) for up to 48 h to promote the accumulation of endogenous GAS5. GAS5 levels increased up to 3.7 fold in cells cultured for 48 h in serum-free medium, and 2.3 fold when cultured in medium containing 0.5% serum compared with cells cultured in the presence of 10% serum (Figure 4A). We have also determined the half-lives of GAS5 with or without serum starvation. Unfortunately, the BRIC method cannot be used for determining the half-life of GAS5 with serum starvation, because the recovery ratio of BrU-RNAs to non-labeled RNAs was too low. We suspect that BrU is not effectively incorporated into serum-starved cells. Instead, we used another inhibitor-free method, 5-Ethynyluridine (EU) pulse-labeling, which is commercially available as the Click-iT Nascent RNA Capture Kit (Invitrogen) [33]–[36]. We have successfully determined the half-life of GAS5 with or without serum starvation by the EU method, and found that *t*_1/2_ of GAS5 was prolonged from 4.2 h to >12 h in serum starved cells (Figure 4B). Similar to the UPF1-depletion experiments, the mRNA levels of cIAP2 and SGK1 were significantly decreased during serum starvation, and those of cIAP2 and SGK1 in medium containing 0.5% serum were slightly higher than those in serum-free medium (Figure 4C). Moreover, the mRNA level of the PPARδ-responsive gene, ADRP, was not significantly changed (Figure 4C). We have also examined whether knockdown of GAS5 suppresses the effects of serum withdrawal on the expression of glucocorticoid-responsive genes. Depletion of GAS5 by RNAi reduced GAS5 RNA levels in HEK293 cells to ∼21.9% of the control level (Figure 5A). The mRNA levels of cIAP2 and SGK1 were not significantly changed during serum starvation in GAS5-depleted cells (Figure 5B). These results indicate that the suppression of apoptosis-related gene expression in response to serum starvation is dependent on GAS5 transcripts.

## Discussion

In this study, we report the importance of stability-mediated ncRNA regulation using GAS5 as a model for long ncRNAs. The UPF1 knockdown experiments showed that GAS5 is specifically degraded by the NMD pathway, and that NMD can regulate the expression levels and the decay rates of GAS5, resulting in a change in the transcriptional activity of several apoptosis-related genes, such as cIAP2 and SGK1 (Figure 2). These effects were rescued by GAS5 over-expression (Figure 3). Moreover, we found that growth arrest by serum starvation elevates the levels of GAS5 and prolonged decay rates, resulting in decrease the levels of apoptosis-related gene mRNAs (Figure 4). These findings are supported by GAS5 knockdown, which suppress the effects of serum starvation on the expression of the apoptosis-related genes (Figure 5). NMD is inhibited by serum starvation because UPF1 phosphorylation is reduced [48]. NMD is also inhibited by other environmental insults, including amino acid starvation, hypoxia, ultraviolet irradiation, other DNA damage-inducing agents, and conditions that activate the unfolded protein response [24], [49]–[51]. Taken together, these observations strongly suggest that certain environmental insults, including serum starvation, inhibit the NMD pathway. This results in an accumulation of GAS5, which causes a suppression of apoptosis-related gene expression, via the GR. To our knowledge, this is the first clear demonstration that the RNA degradation pathway can regulate the function of a long ncRNA in mammalian cells.

Similar to the GAS5 ncRNA, 7SK RNA [52], [53] and steroid receptor RNA activator (SRA) RNA [54], [55] are also long ncRNAs that are involved in transcriptional regulation by binding with transcription factors. 7SK RNA (∼300 bases in human) is transcribed by RNA polymerase III and is located in the nucleus. Together with other associated cellular proteins including hexamethylene bisacetamide-induced 1/2 (HEXIM1/2), 7SK RNA inhibits the activity of the positive transcription elongation factor b (P-TEFb) [56], [57]. P-TEFb is required for RNA polymerase II transcription elongation. The structure of 7SK RNA is conserved across many species [58]. The long 5′ stem–loop that extends from nt 24 to 87 in 7SK RNA binds to the HEXIM1 protein [59], [60] as well as to the 3′ stem–loop from nt 296 to 331 [61]. 7SK RNA is released from P-TEFb/HEXIM/7SK complexes upon an arrest in transcription and in response to physiological stimulations, such as cardiac hypertrophy [62], inhibition of transcription by drugs or UV irradiation [56], [57], [63], which leads to P-TEFb activation.

SRA RNA was first identified in 1999 as a functional ncRNA able to co-activate steroid nuclear receptors [64]. SRA RNA is a long ncRNA that has multiple splice variants. The predominant SRA transcripts in normal tissue are approximately 0.7–0.9 kb while less abundant, larger, transcripts of 1.3–1.5 kb have been identified [54]. SRA RNA is a co-regulator not only of steroid and non-steroid nuclear receptors [64]–[68], but also of several other transcription factors [69], [70]. The SRA RNA folds to form multiple secondary structures, which contribute to its activity [64]. Over-expression of SRA RNA enhances transcription transactivation by steroid receptors. Conversely, knockdown of SRA RNA by siRNA suppressed androgen receptor activity in prostate cancer cells [71]. Many SRA-associated RNA-binding proteins have been found to either positively or negatively regulate the coactivator activity of SRA RNA [68], [69], [72]. Moreover, the SRA1 gene not only produces functional RNAs, but could also lead to the production of a protein [64], [73].

Recently, Yoon et al. reported that lincRNA-p21 stability is changed by the abundance of the RNA-binding protein, HuR, resulting in altered translational activity of a subset of mRNAs [74]. LincRNA-p21 (∼3.1 kb in human) was first noted in 2010 as a repressor in p53-dependent transcriptional responses [75]. In the presence of HuR, lincRNA-p21 is destabilized through the recruitment of let-7/Ago2. HuR then promotes the translation of CTNNB1 and JUNB mRNAs by favoring their association with polysomes. In the absence of HuR, lincRNA-p21 is stable and accumulates, and Rck promotes the association of lincRNA-p21 with CTNNB1 and JUNB mRNAs, repressing their translation through a mechanism that includes a reduction in the size of polysomes. Thus, HuR-dependent translation activation requires rapid degradation of lincRNA-p21 in order to prevent the recruitment of translation repressors onto target mRNAs. This report is particularly important because it shows that an RNA-binding protein can regulate the stability and function of a long ncRNA in mammalian cells.

In addition to the long ncRNAs mentioned above, recent studies have also revealed the functional involvement of long nuclear ncRNAs in the regulation of gene expression (e.g. XIST [76], HOTAIR [77], [78], CDKN2B-AS1/ANRIL [79], [80]) through the modification of chromatin, control of transcriptional and post-transcriptional gene expression (e.g. MALAT1 [37]–[41]), and maintenance of subnuclear structures (e.g. NEAT1/MENε/β [81]–[84]). Interestingly, most of these functional long ncRNAs have short life-spans [7], [17]. We believe that further studies are needed to elucidate the relationships between the RNA degradation pathway and the functions of nuclear long ncRNAs.

## Supporting Information

Figure S1 **Knockdown of UPF1 increases the expression level of GAS5, and decreases the glucocorticoid-responsive genes.** HEK293 cells were treated with a control siRNA or with a siRNA targeting UPF1. The expression levels of indicated genes in control cells (black bar) and in UPF1-depleted cells using UPF1 siRNA (2) (grey bar) was determined by RT-qPCR. The levels of GAPDH and ACTB were used for normalization. Values represent mean±SD obtained from three independent experiments (**P<0.01, student's test).
(TIFF)
