# Supplementary figures and images for: The RNA Degradation Pathway Regulates the Function of GAS5 a Non-Coding RNA in Mammalian Cells
Source: PLoS One. 2013 Jan 30;8(1):e55684. doi: 10.1371/journal.pone.0055684 (PMC3559549; doi:10.1371/journal.pone.0055684)

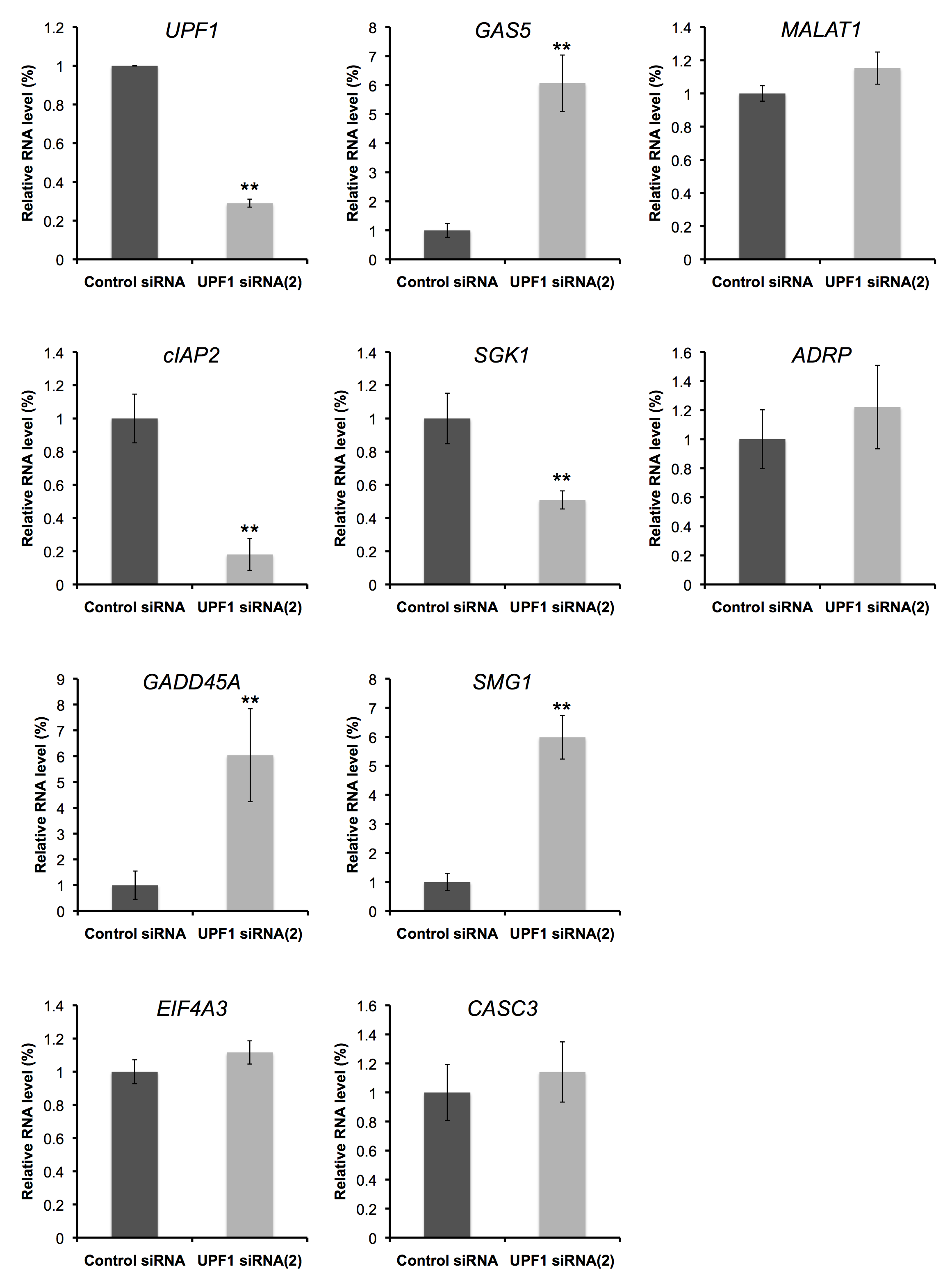

Supplement: Figure S1 — Knockdown of UPF1 increases the expression level of GAS5, and decreases the glucocorticoid-responsive genes. HEK293 cells were treated with a control siRNA or with a siRNA targeting UPF1. The expression levels of indicated genes in control cells (black bar) and in UPF1-depleted cells using UPF1 siRNA (2) (grey bar) was determined by RT-qPCR. The levels of GAPDH and ACTB were used for normalization. Values represent mean±SD obtained from three independent experiments (**P<0.01, student's test). (TIFF) [file pone.0055684.s001.tiff]
